# AI and the Reproduction of Health Inequity: Redistribution-Translation-Accumulation Framework

**DOI:** 10.2196/98158

**Published:** 2026-10-06

**Authors:** Atsushi Nakagomi

**Affiliations:** 1 Department of Social Preventive Medical Sciences, Center for Preventive Medical Sciences, Chiba University, 1-33 Yayoicho, Inage-ku, Chiba-city, Chiba, 263-8522, Japan, 81 43-290-3176

**Keywords:** AI, health inequity, social determinants of health, distributional impact assessment, digital determinants of health

## Abstract

AI is increasingly embedded in the institutions and environments that shape health. Yet current frameworks for understanding its implications for health equity remain underdeveloped. The social determinants of health tradition provides a strong foundation, and recent work on digital determinants of health has begun to address the health implications of digital transformation. However, AI warrants distinct conceptual attention because of its triple role: it operates simultaneously as a determinant of health in its own right, as a mediator and moderator of existing determinants, and as an amplifier of advantage and disadvantage over time. This viewpoint proposes a redistribution-translation-accumulation framework for analyzing how AI may contribute to the reproduction of health inequity. The framework comprises 2 analytically distinct mechanisms and 1 cross-cutting temporal dynamic. Redistribution captures how AI reshapes the distribution of health-relevant resources and opportunities, including education, employment, and income, while AI itself becomes an unequally distributed determinant. Translation describes how AI changes the pathways through which social positions are converted into health outcomes. Proxy-based decision rules can formalize historical inequities, diagnostic algorithms may perform unevenly across populations due to unrepresentative training data, and AI-mediated information environments can alter institutional responsiveness. Accumulation is conceptualized not as a third parallel mechanism but as a temporal amplifier operating on both mechanisms: AI-driven feedback loops and institutional embedding can concentrate advantage and disadvantage over time, often without users’ awareness. The framework is offered as a hypothesis-generating heuristic and is directionally neutral: under specifiable design, deployment, and governance conditions, the same mechanisms can narrow rather than widen health gaps in high-income and low- and middle-income settings alike. The framework has direct implications for governance. Current approaches such as the EU AI Act’s Fundamental Rights Impact Assessment (FRIA) and Canada’s Algorithmic Impact Assessment (AIA) advance AI accountability but assess systems largely before or at deployment and do not systematically track distributional health consequences. Building on this framework, I propose a distributional impact assessment as a complementary tool for equity-oriented AI governance. Structured around the 3 RTA dimensions, it asks whether an AI system alters the distribution of health-relevant resources across groups (redistribution), changes how social positions are converted into health (translation), and risks concentrating disadvantage over time through feedback and institutional embedding (accumulation). It is operationalized with candidate indicators, data sources, responsible actors, and reassessment triggers and is illustrated through a retrospective worked example of a biased care-management algorithm.

## Introduction

AI is increasingly embedded in everyday life. It shapes what people see, think, learn, and do at work; how they seek information; how they are evaluated; which services they can access; and what opportunities are available—often without requiring active “AI use.” Framing the public health question as “does AI help or harm health?” is therefore too narrow. The more consequential issue is how AI enters the social organization of risk, protection, resources, and opportunity that generates population health patterns and unequal health outcomes.

Two clarifications are needed at the outset. First, in this viewpoint, AI refers operationally to computational systems that perform tasks associated with human judgment or decision-making and that are embedded in institutions, platforms, and infrastructures. This definition spans four broad classes of systems that differ in their causal pathways and governance levers: (1) predictive and risk-stratification models; (2) diagnostic and classification systems; (3) automated decision and administrative systems used in welfare, employment, and public administration; and (4) generative models and conversational agents, including large language models and AI companions. The classes are not watertight—generative models, for example, increasingly power administrative and clinical systems—but the first 3 typically operate on people within institutions, whereas the fourth is, at present, most often used directly.

Second, following the World Health Organization (WHO), health inequalities are observable differences in health between population groups, whereas health inequities are the subset of those differences that are unfair, avoidable, and produced by social arrangements rather than by chance or biology alone [1,2]. “Distribution” refers here to how health-relevant resources, opportunities, exposures, and risks—education, income, employment quality, information, institutional responsiveness, and increasingly AI itself—are allocated across population groups. The social determinants of health (SDoH) tradition anchors this inquiry: the WHO describes the SDoH as the conditions in which people are born, grow, live, work, and age, shaped by access to power, money, and resources [1], and major drivers of health inequity are structural conditions—socially produced and potentially modifiable—that distribute advantage and disadvantage across society [3-5].

The central claim of this viewpoint is that AI is best conceptualized not only as a new exposure or a single “digital determinant” but as a socially embedded force that reconfigures how existing determinants are distributed and operate. In doing so, AI can contribute to the reproduction of health inequity—reproducing or amplifying unequal health through institutional embedding and feedback over time. The viewpoint therefore has two aims: (1) to propose a conceptual framework—redistribution, translation, and accumulation (RTA)—for analyzing how AI may contribute to the reproduction of health inequity, and (2) to derive from it a governance instrument, the distributional impact assessment (DIA), that complements existing AI accountability tools. The argument is addressed to public health researchers studying the social production of health inequities, to policymakers and regulators designing AI governance, and to developers and deployers of AI systems. It proceeds in 4 steps: why AI requires an extension of existing SDoH frameworks; how the RTA framework is structured and what it adds; how each component operates, in which direction, and across global contexts; and what follows for governance and research.

## The RTA Framework

### Why AI Requires an Extension of Existing SDoH Frameworks

Classic SDoH models distinguish structural determinants (governance, macroeconomic policies, culture and societal values, and socioeconomic position) from intermediary determinants (material circumstances, behaviors, psychosocial factors, and health system access) [4], and the WHO Commission on SDoH argued that achieving health equity requires action on these upstream drivers [5]. Digital transformation does not merely add a “digital layer” alongside these determinants; it reshapes them across multiple domains. WHO/Europe explicitly frames the digital age as transforming health determinants across digital, social, political, and commercial/economic domains [6], and a subsequent scoping review and expert consensus identified a wide set of determinants that emerged or changed during digital transformation across these domains [7].

This work, however, addressed digital transformation broadly rather than AI specifically. As the review itself notes, “while AI is categorized under the digital determinants of health (DDoH) as a sector-specific policy, a single set of AI policies is unlikely to suffice, especially recognizing the vastly different ways in which AI affects various sectors” [7]. The point matters because the determinants most relevant to health inequity are often those that structure opportunity—education, work, income, institutional responsiveness, information quality—precisely the domains in which AI-mediated systems are increasingly embedded. AI intensifies this transformation as a general-purpose infrastructural layer across institutions and platforms: it can be integrated into educational support [8], workplace management [9], health system interfaces [10], routine everyday interactions [11,12], and allocation and administrative systems [13,14]. AI is thus not only a potential determinant in its own right; it can reorganize how other determinants operate.

### The RTA Framework: Two Mechanisms and a Temporal Amplifier

The challenge is to specify how this broader reorganization of determinants translates into the production and reproduction of health inequity. To avoid an unstructured list of “AI risks,” I propose the RTA framework, comprising 2 analytically distinct mechanisms and 1 cross-cutting temporal dynamic. The 2 mechanisms are redistribution—AI changes how populations are positioned across socially meaningful axes and determinants (who has what)—and translation—AI changes how those determinants and positions are converted into health and well-being (what a given position yields). Accumulation is not a third parallel mechanism but a temporal amplifier that operates on both mechanisms: through feedback loops and institutional embedding, AI-related advantages and disadvantages become concentrated and reproduced over time (Figure 1).

**Figure 1.**
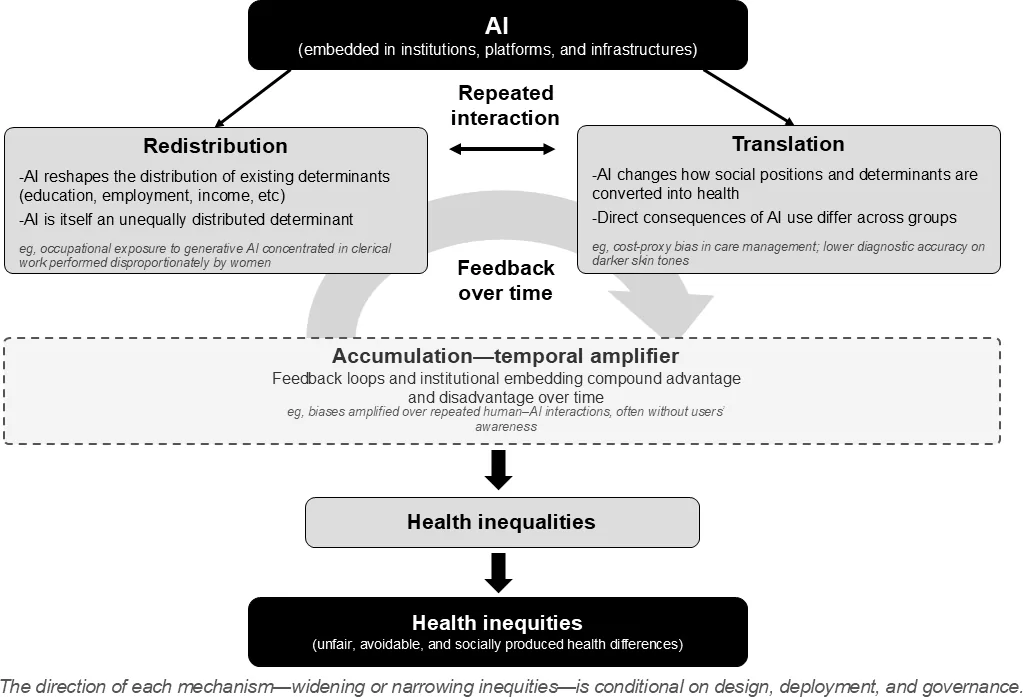
AI and the reproduction of health inequity: the redistribution-translation-accumulation framework.

This architecture requires 3 clarifications. First, the boundary criterion between the 2 mechanisms is the object of change: redistribution concerns changes in the distribution of determinants across groups, whereas translation concerns changes in the health returns to a given determinant or position, holding its distribution constant. The 2 are analytically distinct but not mutually exclusive—a single AI system can do both; the framework is a diagnostic lens, not an exhaustive taxonomy. Second, the components operate simultaneously rather than in sequence: redistribution and translation occur concurrently wherever AI is deployed, and accumulation describes how their effects compound over repeated interactions—with outputs of earlier decisions becoming inputs to later ones. Third, the framework builds on but extends Diderichsen et al’s [15] distinction between differential exposure and differential vulnerability as the 2 mechanisms producing social inequalities in health. There, social stratification determines who is exposed to health-damaging conditions and who is most vulnerable to their effects. AI, however, does not merely add another exposure to an existing order; it intervenes in the stratifying processes themselves—reshaping the distribution of determinants (redistribution), altering the returns to a given position (translation), and formalizing the compounding of both over the life course (accumulation) [16].

The RTA framework is a hypothesis-generating heuristic for structuring research and governance, not an empirically validated causal model. The supporting evidence is heterogeneous—experimental, observational, and audit-based—and much of it concerns intermediate outcomes (judgments, employment exposure, and diagnostic accuracy) rather than measured health outcomes. The population-level magnitude of the hypothesized pathways therefore remains to be established. None of the mechanisms is inherently harmful: each can widen or narrow health gaps depending on design, deployment, and governance.

How the components map onto AI systems is organized less by technical class than by a cross-cutting distinction: people encounter AI either as embedded systems that act on them—allocating, classifying, and filtering, often without any active “use”—or as tools they use directly, of which generative models are currently the most prominent. Each component operates through both modes. Redistribution runs through embedded allocation systems and through unequal access to and capability with directly used tools. Translation runs through the proxies and performance of embedded predictive, diagnostic, and administrative systems and through the socially patterned consequences of direct use—the same tool may be used productively by some and harmfully, or not at all, by others. Accumulation is most salient wherever systems are institutionally embedded and interact repeatedly with the same populations—platforms, administrative systems, and predictive models retrained on their own downstream data. The mapping is not fixed to system class: generative models are themselves increasingly embedded in institutional workflows, at which point they operate like other embedded systems.

### What the RTA Framework Adds Beyond Existing Approaches

The RTA framework differs from 3 adjacent bodies of work in unit of analysis, scope, and explanatory ambition. First, relative to the DDoH framework [6,7], the RTA framework differs in its unit of analysis and mechanism. The DDoH literature catalogs which determinants have emerged or changed during digital transformation; it is an enumerative mapping across domains. The RTA framework instead specifies the generative mechanisms through which a single technology class (AI) acts on the entire determinant structure: it asks not “what are the digital determinants?” but through which processes AI changes, who gets which determinants, what they yield in health, and how those effects compound.

Second, relative to the algorithmic fairness and machine learning for health literature [17-19], the RTA framework differs in scope. That literature has developed sophisticated tools for detecting and mitigating bias within individual models—unequal error rates, proxy bias, and unrepresentative training data—and for incorporating distributive justice into model design [17]. Elements of this model-level practice are being institutionalized in tools such as Canada’s Algorithmic Impact Assessment (AIA) [20], yet they map principally onto one part of an RTA component (translation, within individual systems) and center on system-level and administrative risk. The RTA framework embeds these model-level concerns within a population-health account that also covers AI systems operating far outside health care—in education, labor markets, and public administration—whose health consequences run through the social determinants.

Third, relative to prior conceptual work on AI and health equity, including reviews of AI and health inequities in primary care [21] and ethical machine learning in health care [18], the RTA framework adds an explicit account of AI’s triple role: AI operates simultaneously as a determinant of health in its own right (an unequally distributed resource and exposure), as a mediator and moderator of existing determinants (changing what social positions yield in health), and as an amplifier (compounding advantage and disadvantage through feedback and institutional embedding). This triple role is the fulcrum of the framework’s claim to distinctiveness. Prior frameworks typically address 1 or 2 of these roles; it is their joint operation—and especially the amplification dynamic—that warrants a dedicated analytical framework and a corresponding governance instrument. The amplifier role also carries a governance implication: because accumulation unfolds only after deployment, a framework that centers it must treat postdeployment distributional monitoring as a first-class requirement rather than an afterthought. As elaborated below, neither the Fundamental Rights Impact Assessments (FRIAs) nor the AIA provides this in distributional health terms [20,22]—the gap the DIA is designed to fill.

### Redistribution: AI Reshapes the Distribution of Determinants and Becomes a Determinant That Is Itself Unequally Distributed

AI can reshape the distribution of health-relevant resources and opportunities—how individuals and groups are positioned across key determinants. Among embedded systems, predictive and automated decision systems in hiring, workplace management, and surveillance can shape job quality, support and training, employment trajectories, advancement, and income security [9]. AI may also reshape broader patterns of social stratification. For example, according to the International Labour Organization’s (ILO) refined global index of occupational exposure to generative AI, around one-third of jobs in high-income countries are estimated to have some degree of exposure to generative AI, with clerical occupations continuing to have the highest exposure levels [9]. This matters for inequity because clerical work is often an entry point to relatively stable employment and is disproportionately performed by women. In the same index, jobs in the highest exposure category account for 9.6% of women’s employment compared with 3.5% of men’s employment in high-income countries [9]. Without commensurate redesign of roles, training, and worker protections, task transformation in these occupations may bring reduced job autonomy, wage stagnation, and insecurity—widening gendered and class-based gradients in income, psychosocial stress, and ultimately health [3,9,23].

Through direct use, generative models and conversational agents may support learning, skill acquisition, and educational performance, but these benefits are unlikely to be evenly distributed [8]. Moreover, UNESCO (United Nations Educational, Scientific and Cultural Organization) warns that generative AI can embed bias, exclude minority voices, and perpetuate stereotypes in education, and large language models can reproduce gender and educational stereotypes in generated content [8,24]—influencing who is encouraged toward particular educational or occupational pathways. As access, quality, and effective use vary across groups, AI may create new differences in educational attainment and capability that shape later employment, income, and social mobility.

At the same time, AI itself becomes a determinant of health that can be unequally distributed [25]. Access to high-quality AI tools, reliable infrastructure, safe and culturally or linguistically appropriate AI systems, and the capability to use AI effectively and critically will not be socially neutral: empirical evidence already documents a generative AI divide patterned by personal, positional, and resource-based factors [25]. Nor will the ability to contest automated decisions or to avoid harmful AI-mediated exposures be evenly distributed [26,27]. Redistribution is thus not only about AI changing the distribution of existing determinants; it is also about how the unequal distribution of AI-related resources and protections itself becomes part of the determinant structure.

### Translation: AI Reshapes How Social Positions and Determinants Translate Into Health Outcomes

AI may also change how existing determinants translate into health and well-being: even where social positions remain unchanged, the health returns to education, income, occupation, or social connection may increasingly depend on AI literacy, language compatibility, accessibility, representation in training data, system transparency, and the quality of AI-curated information environments [7,8,10]. Among automated decision and administrative systems, AI may influence who receives timely and appropriate responses in welfare, social services, or health care navigation and whose voices are amplified and needs recognized [13,27]. Risk is greatest when systems operationalize need through proxies that embed historical inequities. In a widely cited example, a predictive care-management algorithm used health care costs as a proxy for health needs, producing systematic racial bias: Black patients had historically incurred lower costs at the same level of illness because of unequal access and spending [28].

Among diagnostic and classification systems, differential performance across populations represents a distinct but related mechanism. Daneshjou et al [29] demonstrated that dermatology AI models performed substantially worse on images of darker skin tones, largely because training datasets disproportionately represented lighter-skinned populations. Unlike proxy-based bias, this form of inequity arises from underrepresentation in training data, producing differential diagnostic accuracy that directly disadvantages underserved groups. This pattern is not unique to AI: the persistence of racial bias in pulse oximetry measurement—in which pulse oximeters overestimate arterial oxygen saturation in Black patients, leading to nearly 3 times the frequency of undetected hypoxemia compared with White patients—shows that differential device performance can remain uncorrected for well over a decade after being documented [30]. The precedent matters for governance: awareness of disparities, without institutionalized measurement and accountability, has historically been insufficient to eliminate them.

For generative models and conversational agents, translation operates through a further pathway: the direct consequences of use may differ across social positions—evidence suggests that the associations between AI companion use and well-being are moderated by users’ social connectedness and loneliness [31]. Two clarifications sharpen this mechanism. First, differential performance is distinct from globally poor performance: a model equally poor for all groups presents an accuracy problem; translation concerns performance, responsiveness, or fit that differ systematically across social positions [17,19]. Second, these processes may operate through multiple pathways—psychosocial stress and agency, affordability and accessibility of services, exposure to misinformation, and institutional responsiveness [10,24,27,31]. The same structural position may yield different health consequences depending on how AI-mediated systems are designed, configured, and governed.

### Accumulation: AI Amplifies Advantage and Disadvantage and Reproduces Them Over Time

Health inequity is rarely produced by one-off exposures; it is generated through cumulative processes in which advantage and disadvantage compound [16,32]. Accumulation is the temporal dynamic through which the effects of redistribution and translation are amplified over time. It operates through 2 channels corresponding to the 2 modes of encounter: institutional embedding in organizational routines and feedback loops in repeated human-AI interaction.

Regarding the first channel, small initial differences in access, capability, or representational inclusion can translate into persistent differences in system performance, institutional response, and opportunity. Better-positioned groups may receive better system fit, greater benefits from AI-enhanced services, and more capacity to contest errors, while disadvantaged groups may experience repeated misclassification, lower-quality outputs, and more barriers to redress [7,10]. Once embedded into organizational routines, such differences can become durable. A related dynamic operates at the data level: when an embedded system’s outputs shape who receives services, and service records become training data for subsequent updates, initial disparities are written into successive generations of the system [28,33].

Regarding the second channel, Glickman and Sharot [34] demonstrated experimentally that human-AI feedback loops can systematically alter human perceptual, emotional, and social judgments, with biases amplifying over successive interactions. Critically, participants were largely unaware that their judgments had shifted—AI-driven bias amplification may operate below the threshold of conscious recognition. This has direct implications for health inequity. If such loops alter risk perceptions, health-seeking behavior, or clinicians’ assessments of patient need, the resulting biases may accumulate without detection or correction [27,34]. Across both channels, accumulation is best understood as operating on the other 2 components: it takes the unequal distributions produced by redistribution and the unequal returns produced by translation and converts them into persistent, self-reinforcing patterns.

### Direction Is Not Destiny: Conditions Under Which AI May Narrow Health Gaps

An analytical lens must be directionally neutral: each RTA component can, under specifiable conditions, narrow rather than widen health inequities—indeed, the argument that AI could contribute to the reproduction of health equity can be made through the same 3 components. Through redistribution, AI can extend scarce expertise. The counterfactual against which AI systems should be judged is often not perfect human care but, rather, no care: an estimated 3 billion people worldwide lack adequate access to dermatological care, so even an imperfect AI triage tool could expand effective access if its performance is adequate across skin tones [29]. Notably, the same study demonstrated the remedy: fine-tuning models on diverse, curated images closed the performance gap between light and dark skin tones [29]. AI tutoring and translation tools could similarly benefit those facing language, literacy, or geographic barriers, if designed for accessibility and deployed with intent [8,35].

Through translation, systems can be designed to weaken, rather than reproduce, the link between social position and health. Rajkomar et al [17] argue that machine learning systems should be used proactively to advance health equity by incorporating principles of distributive justice into model design, deployment, and evaluation. Concretely, this includes replacing biased proxies with more direct measures of need—the remedy demonstrated in the Obermeyer et al [28] case, where reformulating the prediction target reduced the racial bias by 84%—curating representative training data and evaluating subgroup performance as a condition of deployment.

Through accumulation, the same feedback dynamics can be harnessed in reverse: early detection of emerging subgroup disparities, fed back into system redesign, makes equity improvement self-reinforcing [10,17]. The direction each mechanism takes is therefore conditional, not intrinsic. The key conditions are whether training data represent the populations served; whether systems are accessible across language, literacy, disability, and connectivity gradients; whether deployment prioritizes disadvantaged settings or follows purchasing power; whether affected groups can contest decisions; and whether subgroup performance is monitored after deployment [17,29,35,36]. These conditions are precisely what the governance instrument proposed below is designed to assess.

### The RTA Framework in a Global Context

The evidence and governance instruments discussed so far are drawn largely from high-income countries, and this partiality must be acknowledged: health inequity is overwhelmingly a global phenomenon, and the RTA mechanisms are likely to operate differently—and often more forcefully—in low- and middle-income countries (LMICs). Most AI-based health applications are developed in high-income countries, and their transfer to LMIC contexts is relatively recent and lacks robust local evaluation [37]. In RTA terms, redistribution operates through infrastructure and workforce channels: unequal infrastructure, connectivity, and AI-related skills may concentrate AI’s benefits in already-advantaged regions, while occupational exposure to automation differs across income levels [9]. Translation operates through representation: systems trained on data drawn predominantly from high-income, English-speaking, and digitally connected populations may perform worse for the global majority [37], and scholars of data colonialism argue that the extraction of data from populations who have little say in, or benefit from, its use reproduces older patterns of appropriation [38]. Accumulation operates through governance capacity: where the expertise and infrastructure for AI governance and monitoring are underresourced [37], disparities and harmful feedback loops are less likely to be detected and corrected and become entrenched. The direction-is-conditional argument also applies globally: AI could extend scarce specialist capacity where systems are locally validated, adapted, and governed [37]. LMIC settings are therefore not an afterthought but the contexts where both the risks and the potential equity gains are largest.

## From Framework to Governance: Implementing the DIA

### The Gap in Existing Governance Tools

If AI can reshape the distribution of determinants, change how they translate into health, and compound the results over time, governance cannot focus only on average accuracy or innovation benefits. Equity-oriented governance requires attention to distributional impacts: who benefits, who is excluded, whose needs are represented, what proxies are used, and whether harms accumulate disproportionately among already disadvantaged groups [17,21,28]. The WHO’s recent guidance on large multimodal models for health emphasizes the need for robust ethics and governance across the AI lifecycle, including evaluation beyond predeployment testing [10]. UNESCO similarly emphasizes human rights, fairness, nondiscrimination, transparency, accountability, and human oversight [39].

Emerging regulatory frameworks have begun to address some of these concerns. The EU Artificial Intelligence Act [40] requires deployers of certain high-risk AI systems to conduct FRIAs, which evaluate risks to fundamental rights, including nondiscrimination and access to essential services [22]. Canada’s AIA provides a structured questionnaire for federal agencies to assess automated decision systems before deployment, with scheduled reviews thereafter [20]. These are important advances, yet they do not systematically evaluate how AI systems may redistribute health-relevant resources, alter the translation of social positions into health outcomes, or generate cumulative disadvantage over time. The pulse oximetry precedent underscores the stakes: documented disparities can persist for many years when no institution must measure them, act on them, or answer for them [30].

### A DIA Structured Around the RTA Dimensions

To address this gap, I propose a DIA structured around the RTA dimensions as a complementary governance tool. This proposal builds on the tradition of health impact assessment (HIA)—procedures, methods, and tools used to judge a policy’s potential effects on population health and their distribution [41]—and its equity-focused extensions (EqHIA), which assess how proposals may affect health inequities across groups [42,43]. Reviews, however, consistently find equity inadequately addressed within HIA—owing to limited guidance, definitions, data, and methods [43]—and a recent systematic review concludes that approaches remain diverse and fragmented [44]. This underoperationalization is part of the motivation for a mechanism-anchored instrument. Moreover, HIA and EqHIA are applied primarily as prospective, single-point tools, whereas redistribution and translation unfold—and accumulation compounds them—through repeated interactions and institutional embedding over time; the DIA must therefore function as both a predeployment requirement and a framework for ongoing postdeployment monitoring.

At its core, the DIA asks evaluators three questions: (1) redistribution—does the AI system alter the distribution of health-relevant resources, opportunities, or exposures or create or widen inequalities in access to AI-related benefits and protections? (2) translation—does it change how social positions are converted into health outcomes—through proxies, differential performance, or altered information environments—or do the direct consequences of use differ across groups? (3) accumulation—do the system’s feedback loops, institutional embedding, or interaction dynamics risk concentrating disadvantage over time in ways that may be difficult to detect or reverse? For the DIA to be more than EqHIA with an AI label, these questions must be operationalized. Table 1 outlines candidate indicators and evidence types, data sources, responsible actors, and lifecycle timing for each dimension. Three operational features distinguish the DIA from existing practice. First, unlike EqHIA, which typically assesses a policy prospectively at a single decision point [42,43], the DIA is anchored to the AI life cycle, with defined reassessment triggers: model updates or retraining, expansion to new populations or settings, drift in input data or user composition, and breach of prespecified subgroup performance thresholds. Second, unlike technical algorithmic audits, which evaluate model-level fairness metrics at a point in time [45], the DIA takes population health distribution as its outcome and extends to nonclinical AI—educational, occupational, administrative—whose health effects operate through the social determinants. Third, unlike FRIA and AIA, organized around rights and administrative risk, respectively [20,22]—and whose updates track changes in the system rather than observed distributional outcomes—the DIA is organized around the mechanisms by which health inequity is socially produced, making cumulative and feedback effects assessable rather than invisible.

**Table 1.** Operationalization of the distributional impact assessment across the redistribution-translation-accumulation (RTA) dimensions.

RTA dimension	Core question	Candidate indicators and evidence types	Data sources	Responsible actors and lifecycle timing
Redistribution	Does the system alter the distribution of health-relevant resources, opportunities, and exposures across groups—including access to AI itself?	Changes in the distribution of employment, education, income, and service allocation across sociodemographic groups; access to and use of AI systems by group; availability of contestation and opt-out mechanisms across groups	Administrative data on employment, education, income, and service allocation; deployment and usage logs; population surveys of access, use, and AI-related capabilities	Deployer, with independent review proportionate to risk; academic researchers for population-level access and capability analyses; predeployment projection, then periodic postdeployment reporting
Translation	Does the system change how social positions are converted into health outcomes (proxies, differential performance, and altered information environments), and do the direct health consequences of AI use differ across groups?	Group differences in the health and well-being returns to determinants (eg, education, income, and employment) conditional on AI exposure; group differences in the direct effects of AI use on health and well-being; proxy audit: what the target variable measures and for whom; subgroup performance (calibration, false positive and false negative rates); accessibility across language, literacy, and disability	Independent longitudinal panel or cohort data linking social position, AI exposure and use, and health and well-being outcomes; training data documentation and datasheets; independent validation studies on representative data; user testing with affected groups	Developer jointly with deployer for predeployment; independent auditors for high-risk systems; academic researchers for population-level analyses of differential health returns; reevaluation on model update
Accumulation	Do feedback loops, institutional embedding, or interaction dynamics risk concentrating disadvantage over time?	Trends in subgroup disparities across successive model updates; drift in input data and user composition; whether system outputs feed back into future training data; patterns of complaints and redress by group	Longitudinal monitoring dashboards; model update and retraining logs; public registries of high-risk systems; community reporting channels	Deployer with regulatory oversight, in collaboration with academic researchers for longitudinal disparity analyses; continuous postdeployment monitoring, with defined reassessment triggers (model updates, data or user drift, subgroup threshold breaches, and expansion to new populations or settings)

Predeployment DIAs would most naturally be conducted by deploying organizations—health systems, education authorities, employers, and public agencies—with independent review proportionate to risk, mirroring the FRIA’s deployer obligations [22]. Deployer self-assessment cannot carry the whole instrument, however: estimating group differences in health returns conditional on AI exposure, or longitudinal subgroup disparity trajectories across model updates, requires research-grade longitudinal data and methods that deployers rarely hold, presupposing collaboration with academic researchers whose independent analyses audit deployer-led assessments. Postdeployment monitoring additionally requires that deployers bear the cost of routine subgroup reporting as a condition of procurement or reimbursement, that affected communities have standing to trigger reassessment, and that findings feed into the public registries where high-risk systems are already registered [22]. Stakeholder adoption is itself a distributional variable the DIA should track: which organizations adopt a system first, which professionals and users take it up, and who is left out will shape benefits and harms independent of the system’s technical properties [35,36]. Distributional goals can also conflict: impossibility results show that plausible fairness criteria cannot generally be satisfied simultaneously when base rates differ across groups [19,46]. The DIA therefore does not prescribe a single fairness metric; it requires evaluators to state and publicly justify which distributional criteria are prioritized in a given deployment context.

### A Retrospective Worked Example: Applying the DIA to a Care-Management Algorithm

The DIA’s value is best illustrated by applying it retrospectively—following the indicators, data sources, actors, and timing in Table 1—to the care-management algorithm analyzed by Obermeyer et al [28]. For redistribution, the deploying health system should have examined, before deployment and using its own claims data, how program enrollment would be distributed across sociodemographic groups relative to clinical need. Because the algorithm allocated scarce slots across a large insured population, it plainly redistributed a health-relevant resource. Yet a predeployment projection cannot settle the question: even with equal allocation, actual uptake could be unequal because enrollment depends on contact, trust, language, and navigation capacity that are socially patterned. The realized distribution is observable only after deployment, so periodic reporting of enrollment composition by group against clinical need is a core DIA output.

For translation, the developer, jointly with the deployer and under independent audit, should have conducted a predeployment proxy audit: what does the target—predicted health care costs—measure and for whom? Subgroup calibration on linked claims and clinical data would have surfaced the core problem: the cost proxy systematically understated Black patients’ need [28]. The same audit would have identified the remedy the authors demonstrated—reformulating the prediction target toward direct health measures, which reduced the bias by 84% [28]. Assessment cannot end there, however: calibration degrades as populations and proxies drift [47], so it should be repeated on live data at every model update, with subgroup thresholds as reassessment triggers.

For accumulation, the deployer, under regulatory oversight and with academic researchers, should have monitored subgroup enrollment across updates and documented whether outputs fed back into training data. The risk is concrete: patients screened out receive less proactive care, generating lower costs that confirm the algorithm’s assessment in retraining [28,33]—an entrenchment dynamic invisible to a one-off audit. A prespecified divergence threshold would have detected, as routine DIA output, the disparity that instead required independent researchers with unusual data access to uncover. None of the 3 questions requires novel science; what was missing was an institutional requirement specifying what should be examined, by whom, and when.

## A Research Agenda

A practical research agenda follows from this framing. First, researchers should assess how AI changes the distribution of health-relevant resources and opportunities, including the unequal distribution of AI itself [25]. Second, studies should identify how AI modifies the mechanisms linking determinants to health and well-being, attending to heterogeneous effects and to the conditions under which effects run in an equity-improving direction [31]. Third, longitudinal and institutional analyses are needed to understand how AI-related advantages and disadvantages accumulate and become reproduced over time through feedback loops and organizational embedding. Fourth, the agenda should be globally scoped, prioritizing locally grounded evaluations in LMIC settings [37]. The goal is to move from “AI effects on health” toward “AI reshaping the social production of health inequity.”

## Conclusion

AI should be treated not merely as a new exposure but as a socially embedded force that can reconfigure the generative processes underlying health inequity. The RTA framework advanced here provides an analytical lens: 2 mechanisms—redistribution of health-relevant determinants and translation of social positions into health—compounded by accumulation, a temporal amplifier operating through feedback and institutional embedding. Current governance instruments do not yet address the distributional health consequences of AI systematically; a DIA, structured around the RTA dimensions and operationalized through concrete indicators, actors, and reassessment triggers, could help bridge this gap. The central task for public health research and governance is not only to estimate whether AI helps or harms health on average but also to examine how AI enters the generative processes that produce health inequity—and to ensure that governance prevents inequity from being scaled and automated.

## Abbreviations

AIA: Algorithmic Impact Assessment
DDoH: digital determinants of health
DIA: distributional impact assessment
EqHIA: equity-focused health impact assessment
FRIA: Fundamental Rights Impact Assessment
HIA: health impact assessment
ILO: International Labour Organization
LMIC: low- and middle-income countries
RTA: redistribution-translation-accumulation
SDoH: social determinants of health
UNESCO: United Nations Educational, Scientific and Cultural Organization
WHO: World Health Organization
